# Examining the Long-term Association Between Neighborhood Socioeconomic Status and Obesity and Obesity-related Unhealthy Behaviors Among Children: Results From the Fragile Families and Child Wellbeing Study

**DOI:** 10.1093/abm/kaad001

**Published:** 2023-03-31

**Authors:** Yeonwoo Kim, Yue Liao, Natalie Colabianchi

**Affiliations:** Department of Kinesiology, University of Texas at Arlington, Arlington, TX, USA; Department of Kinesiology, University of Texas at Arlington, Arlington, TX, USA; School of Kinesiology, University of Michigan, Ann Arbor, MI, USA

**Keywords:** Neighborhood socioeconomic status, Childhood obesity, Obesity-related unhealthy behaviors, Sedentary behaviors, Neighborhood effects on child health

## Abstract

**Background:**

Literature has focused on neighborhood environments and their possible impacts on obesity and obesity-related behaviors. However, few longitudinal studies have examined the effect of neighborhood socioeconomic status (nSES) on childhood obesity.

**Purpose:**

Investigate the longitudinal association between nSES and obesity and obesity-related unhealthy behaviors.

**Methods:**

We obtained data from the Fragile Families and Child Wellbeing Study (*N* = 2,072). The main exposure was nSES (measured using an index of five variables representing wealth, income, education, and occupation from the Decennial Census 2000) at ages 3, 5, and 9. The outcome was children’s body mass index *z*-score (BMIz) at ages 5, 9, and 15. Three measures of obesity-related behaviors (i.e., child- or caregiver-reported soda/snack food intake, fast-food intake, and sedentary behaviors) at ages 5, 9, and 15 were included as mediators and outcomes. Cross-lagged path analyses were conducted.

**Results:**

Higher nSES at a previous wave was associated with consuming less soda/snack foods (*β*s = −0.15 to −0.11 [varying by ages], *p* < .05) and fast-food intake (*β*s = −0.21 to −0.14 [varying by ages], *p* < .01), and less frequent sedentary behaviors (*β*s = −0.14 to −0.06 [varying by ages], *p* < .01), but not with BMIz (*β*s = −0.08 to 0.05 [varying by ages], *p* > .05). Unhealthy behaviors did not mediate the nSES–BMIz association at alpha .05.

**Conclusion:**

Health policies need to target low-socioeconomic neighborhoods to shape healthy lifestyles in children. To develop effective interventions, future research needs to examine comprehensive potential mediators like obesity-related parenting skills, home environments, and built and social environments on the risk of childhood obesity and obesity-related behaviors.

## Teaser

Children who resided in socioeconomically disadvantaged neighborhoods during early childhood had unhealthier behaviors from middle childhood through adolescence compared to those in advantaged neighborhoods.

## Introduction

Childhood obesity is a serious health condition in the United States (U.S.). The prevalence of overweight and obesity among children and adolescents has been increasing in the past decades [1–3]. The latest data from the National Health and Nutrition Examination Survey indicate that an estimated 19.3% of U.S. children and adolescents have obesity, and another 16.1% are overweight [3]. Excessive weight puts children at a greater risk for type 2 diabetes mellitus, hypertension, nonalcoholic fatty liver disease, obstructive sleep apnea, and dyslipidemia—conditions that have been previously more common in adults [4]. Further, childhood obesity is associated with adulthood obesity, metabolic syndrome, diabetes, and cardiovascular diseases [5–8]. In addition to its negative impact on physical health, childhood obesity is also associated with societal weight stigmatization, weight bias, and discrimination [9–11], which consequently can result in psychological comorbidities, such as depression, emotional and behavioral disorders, and low self-esteem [12]. Thus, childhood obesity is considered one of the most serious public health challenges that we are currently facing.

Factors that influence childhood obesity are likely multifaceted, including interactions between genetic, biological, psychological, sociocultural, behavioral, and environmental factors [13, 14]. In particular, the increasing prevalence of childhood obesity over the past decades is believed to be a result of increased energy content of diet, decreased level of physical activity, and increased sedentary lifestyle [15–17], which can be attributed to dramatic societal changes, such as use of screen-based media, decrease in active commuting, and insufficient school physical education. There have been numerous efforts to help children and adolescents with healthier behavioral changes through lifestyle interventions; however, the upward trend of childhood obesity and unhealthy lifestyle at the national scale has not slowed down [2, 3]. Therefore, it is important to consider how the larger context of where children live and grow up might impact obesity-related unhealthy behaviors and develop multilevel strategies to combat childhood obesity.

Neighborhood environment has been well-recognized as one of the determinants that are linked to childhood obesity. In 2005, the Institute of Medicine [18] used the Social Ecological Model which highlights factors that are beyond individual-level characteristics and behaviors to describe the etiology of the childhood obesity epidemic and since then, this theoretical framework has been used to inform many obesity interventions. Health outcomes such as obesity, are influenced by individuals’ interactions with the larger economic contexts in which they live [19–21]. According to the neighborhood resource model, unfavorable neighborhood socioeconomic environments (e.g., census tract-level percentage of individuals without a high school diploma, poverty rates) are believed to influence children’s obesity-related behaviors and weight status possibly through accessibility to health-related resources (e.g., large grocery stores, fast-food restaurants, physical activity facilities, bike lanes, parks, and playgrounds) within neighborhoods [22–27]. One study showed that children living in low-socioeconomic neighborhoods engaged in more sedentary behaviors compared to those living in higher-socioeconomic neighborhoods [26]. Another study showed that the presence of affluent neighbors was positively associated with healthy eating and negatively associated with both unhealthy eating and screen time [23]. A study using data from the National Survey of Children’s Health showed that children living in the least favorable socioeconomic neighborhood were more likely to be overweight and obese, and this increased risk was partly explained by differences in behavioral risk factors (i.e., sedentary behavior and physical activity) [24].

Despite the growing interest in the neighborhood effects on obesity, a limited number of studies have investigated the longitudinal impact of neighborhood socioeconomic status (nSES) on childhood obesity and associated unhealthy behaviors [25, 26, 28–33]. A systematic review [34] identified that among 33 peer-reviewed studies, five longitudinal studies investigated the nSES impact on childhood obesity or physical activity, and none investigated the longitudinal effect of nSES on children’s eating behaviors. For example, Pabayo et al.[28] reported that neighborhood socioeconomic deprivation was associated with lower physical activity levels at age 10, and the neighborhood effect persisted at ages 11 and 15 years. Further, within the limited longitudinal studies, very few measured time-varying nSES to account for participant moves and changes in nSES conditions over time [25, 29, 30]. In addition, few studies have examined if the nSES impact on children’s weight status is mediated by children’s obesity-related behaviors [35–37], although an imbalance between caloric intake and expenditure is recognized as the most immediate non-genetic determinant of childhood obesity [38]. To our knowledge, no study has investigated the long-term effects of nSES as a time-varying exposure on childhood obesity and obesity-related behaviors from early childhood to adolescence and the behavioral mechanism by which nSES might impact childhood obesity longitudinally.

This study aims to examine the lagged, longitudinal association between nSES and childhood BMI *z*-score and obesity-related unhealthy behaviors (measured by soda/snack food intake, fast-food intake, and sedentary behaviors of children) from early childhood (age 5) to adolescence (age 15) and test the mediating role of obesity-related unhealthy behaviors in the association between nSES and child BMI *z*-score. As the prevalence of obesity has almost doubled from young childhood (2- to 5-years old) to adolescence (12- to 19-years old) [1, 2], this investigation can provide evidence to support policy formulation and intervention development that targets certain neighborhoods and addresses the health disparities in childhood obesity.

## Methods

### Data

Data were extracted from the Fragile Families and Child Wellbeing Study (FFCWS) to examine the long-term association between nSES and children’s BMI *z*-score and obesity-related unhealthy behaviors. The FFCWS is a national, ongoing longitudinal birth cohort survey of 4,898 children born in 1998–2000 and their parents drawn from a stratified sample of 20 U.S. large cities [39]. The FFCWS uses a stratified random sampling approach from all cities with populations of 200,000 or more and oversampled unmarried parents and African American families. The FFCWS collected data about demographic status, socioeconomic status, and child health and well-being when the child was born (1998–2000) and at ages 1 (1999–2001), 3 (2001–2003), 5 (2003–2006), 9 (2007–2010), and 15 (2014–2017) years old. Also, the FFCWS provides restricted-use census tracts data which include census tract-level demographic and socioeconomic information from the 2000 Decennial Census. Data from the Decennial Census 2000 were linked to the FFCWS data from baseline and the 1-, 3-, 5-, and 9-years studies. Further details about the FFCWS’s sample designs have been described elsewhere [39]. The present study uses data from the 3-, 5-, 9-, and 15-years studies linked to census tract data based on respondents’ residential addresses. Our analytic sample is 2,072 children whose anthropometric measurements were taken at least twice during the follow-up studies because our study focuses on changes in child BMI *z*-score over time. Children who dropped out were more likely to be Hispanic and have a lower level of parental highest educational attainment compared to those who remained in the study. There were no significant differences in child sex, household income-to-needs ratio, time-varying neighborhood socioeconomic status, time-varying child BMI, time-varying soda/snack food intake, time-varying fast-food intake, and time-varying sedentary behaviors.

### Measures

Neighborhood socioeconomic status (nSES). nSES was calculated for three time points when the child was at ages 3, 5, and 9. nSES was defined as the weighted average of socioeconomic characteristics in a census tract where the focal child lived at each data collection point. We selected five variables representing wealth and income (median household income; median value of housing units), education (the percentage of adults 25 years of age or older who had completed high school; the percentage of adults 25 years of age or older who had completed college), and occupation (the percentage of employed persons 16 years of age or older), which were utilized to create nSES in past literature [40, 41]. nSES score for each time point (i.e., at ages 3, 5, and 9) was calculated by summing the *z*-scores of these five variables and then standardizing it within the FFCWS full sample, with greater values indicating more advantageous socioeconomic conditions.

Body mass index *z*-score. One of our outcome measures was age- and sex-specific body mass index *z*-score (BMIz) when children were 5, 9, and 15 years of age. Age- and sex-specific BMIz is a traditionally used standardized measurement in childhood obesity literature [42]. Child height and weight were measured twice in the child’s home at each data collection, and a third measurement was taken if the weight difference between the first two measurements was 2 pounds or more or if the height difference was 2 cm or more. BMIz was computed based on the CDC’s growth chart. Anthropometric measurements with implausible measurements or missing measurements were set to missing. Additional details on anthropometric measures are available on the FFCWS website (fragilefamilies.princeton.edu).

Unhealthy Diets. We examined soda/snack food intake and fast-food intake at ages 5, 9, and 15 as mediators and outcome measures. Soda and snack food intake at ages 5 and 9 were measured based on caregiver-reported number of servings of soda, candy, sweets, snack food, or chips the child eats on a typical day. For age 15, soda intake was measured by child-report on the response to the question, “In a typical week, how many regular, non-diet sweetened drinks do you have?” Snack intake was not measured at age 15 in the original FFCWS. Fast-food intake at ages 5, 9, and 15 was examined by asking how often the child eats a meal from a fast-food restaurant to primary caregivers at ages 5 and 9 and children at age 15. To allow for comparability among different measurements in ages 5, 9, and 15 in a multivariate analysis, these measures were standardized to have a mean of 0 and a standard deviation of 1 within the full sample of the FFCWS, with greater values indicating greater unhealthy diets.

Sedentary Behaviors. We examined caregiver-reports of sedentary behaviors at age 5 and child-reports of sedentary behaviors at ages 9 and 15 as a mediator and an outcome measure. For age 5, sedentary behaviors were examined by summing the primary caregiver’s responses to the questions assessing the number of hours of watching television and playing computer games or video games on their weekday and weekend. Sedentary behaviors at age 9 were examined by summing the child’s responses to the questions about their weekday and weekend frequency of computer time doing schoolwork and chatting/instant messaging with friends. For age 15, sedentary behaviors were examined by summing the child’s response to the questions assessing the number of hours of playing games on the computer, TV, or a handheld device, visiting websites or shopping on the Internet, and watching TV, videos, and movies on weekdays. To allow for comparability among different measurements at ages 5, 9, and 15 in a multivariate analysis, these sedentary behaviors measures were standardized to have a mean of 0 and a standard deviation of 1 within the full sample of the FFCWS, with greater values indicating greater sedentary behaviors.

Covariates. Covariates include child sex (boy, girl), child-reported race/ethnicity (non-Hispanic White only, non-Hispanic Black/African American only, Hispanic, non-Hispanic other), highest parental education attainment at age 3 (less than high school, high school graduate and equivalent, some college, and college graduate and above), household income-to-needs ratio at age 3 (0–49%, 50–99%, 100–199%, 200–299%, and 300% and above), and child BMIz at age 3. Child race/ethnicity was self-reported at age 15. All sociodemographic characteristics are time-invariant measures.

### Analysis

**Fig. 1.**
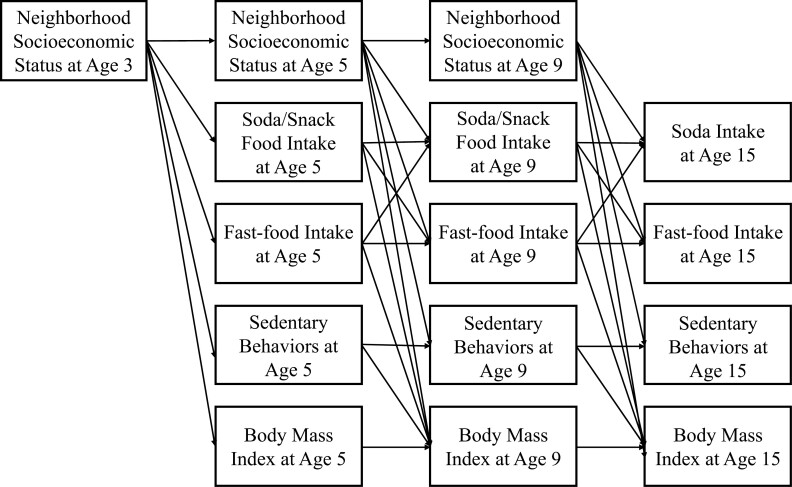
Initial model indicating a hypothesized behavioral mechanism between neighborhood socioeconomic status and child body mass index *z*-score. Child sex, race/ethnicity, parental education level, and household income-to-needs ratio were controlled, and covariates pathways are not depicted for simplicity.

We conducted a cross-lagged path analysis based on a hypothesized model shown in Fig. 1. A cross-lagged path analysis estimates the stability (i.e., autoregressive relationship) and relationships between variables over time (i.e., cross-lagged relationship) using longitudinal data. An autoregressive relationship is an association between each observation and the observation preceding it (e.g., BMIz at age 5 and BMIz at age 9), and a cross-lagged relationship indicates an association between two repeated measures (e.g., nSES at age 3 and BMIz at age 5).

We built cross-lagged path models sequentially by testing path invariance and error covariance invariance in a series of models, to determine the final optimal model based on the coefficient of determination across models with the model simplicity principle. The best-fit model included constrained autoregressive paths for BMIz and nSES and constrained cross-lagged paths from nSES to sedentary behaviors and from sedentary behaviors to BMIz. We estimated indirect effects by multiplying the direct path coefficients between variables of interest. In addition, because a traditional cross-lagged path model had a better model fit than a random intercept cross-lagged path model (standardized root mean square residual [SRMSR]: 0.050 vs. 0.096), a random intercept was not considered further. Of note, a smaller SRMSR indicates a better model fit [43].

We used a full-information procedure, a method of deriving likelihood functioning based on all available data for imputation [44]. We confirmed that three-quarters of cases had non-missing or 1–2 missing values for all 20 variables of interest. We did not conduct a multilevel analysis at the census tract-level because 94% of census tracts had one or two participating children and the intra-class correlation coefficient (ICC) is low (ICC < 0.02) given the use of multilevel modeling is justified when ICC is greater than 0.05 [45]. The complex survey design of the FFCWS was accounted for; thus, our estimates are representative of the originally sampled cities. All analyses were conducted in StataSE 16 and MPlus 8.

## Results

### Descriptive Statistics

Table 1 provides sample characteristics. The sample consisted of 2,072 children, including 1,062 boys (51%) and 1,010 girls (49%). As shown in Table 1, 44% of children self-reported their race/ethnicity as non-Hispanic Black/African American only, followed by Hispanic (21%) and non-Hispanic White only (18%). More than a third of children lived in households in poverty at age 3. Seventeen percent of children were overweight or obese at age 3, and this increased to 24% at age 15. About 48% of children in our analysis moved at least once from ages 3 to 9, and among those that did move, 62% experienced a change in neighborhood SES (results not shown).

**Table 1:** Sample Characteristics (*N* = 2,072)

Characteristics	Percentage
Child sex	
Boys	51.3%
Girls	48.8%
Child race/ethnicity	
Non-Hispanic White only	18.3%
Non-Hispanic Black/African American only	43.8%
Hispanic	21.0%
Non-Hispanic other	6.9%
Missing	10.0%
Parental highest education level at age 3	
Less than high school	13.9%
High school graduate and equivalent	27.7%
Some college	41.3%
College graduate and above	17.1%
Household income-to-needs ratio at age 3	
0–49%	20.2%
50–99%	19.0%
100–199%	24.5%
200–299%	14.4%
300% and above	21.1%
Missing	0.9%
Overweight/obese at age 3 (body mass index *z*-score ≥1.64)	17.3%
Overweight/obese at age 5 (body mass index *z*-score ≥1.64)	16.2%
Overweight/obese at age 9 (body mass index *z*-score ≥1.64)	23.5%
Overweight/obese at age 15 (body mass index *z*-score ≥1.64)	24.1%
Number of servings of soda/snack food intake at age 5 per day (*M* ± SD)	3.78 ± 2.77
Number of servings of soda/snack food intake at age 9 per day (*M* ± SD)	3.19 ± 2.47
Number of soda drinks at age 15 per day (*M* ± SD)	2.29 ± 2.00
Number of fast-food meals at age 5 per week (*M* ± SD)	2.25 ± 1.02
Number of fast-food meals at age 9 per week (*M* ± SD)	1.19 ± 0.99
Number of days of fast-food intake at age 15 (*M* ± SD)	1.77 ± 1.41
Hours of sedentary behaviours at age 5 per day (*M* ± SD)	7.88 ± 5.15
Frequency of sedentary behaviours at age 9 per day (Likert scale) (*M* ± SD)	5.28 ± 0.72
Hours of sedentary behaviours at age 15 per day (*M* ± SD)	9.74 ± 6.54

*M* indicates mean, and SD indicates standard deviation. Child race/ethnicity was self-reported at age 15. Frequency of sedentary behaviours at age 9 was calculated by summing the child’s responses to four questions rated via a 4-point Likert scale (1 = half of hour or less per weekday; 2 = more than half an hour but less than an hour per weekday; 3 = 1–2 hours per weekday; 4 = more than 2 hours per weekday).

### Model Testing

**Fig. 2.**
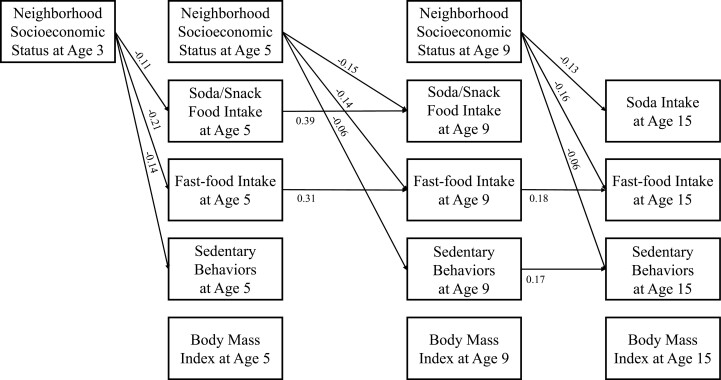
Path model with significant standardized estimates of autoregressive and cross-lagged relationships of our interest. While Table 2 presents unstandardized estimates, this figure presents standardized estimates. Insignificant autoregressive and cross-lagged relationships were not presented for simplicity. Child sex, race/ethnicity, parental education level, and household income-to-needs ratio were controlled, and covariates pathways are not depicted for simplicity. Neighborhood socioeconomic status, soda/snack food intake, soda intake, fast-food intake, and sedentary behaviors were standardized to have a mean of 0 and a standard deviation of 1 within the full sample of the FFCWS. The analysis accounted for the complex survey design of the FFCWS.

Figure 2 presents the results of the path model with standardized estimates of significant cross-lagged relationships. The coefficient of determination for the final model was 0.89, which is considered as strong [46]. Higher nSES at age 3 was associated with consuming less soda/snack foods (*β* = −0.11, *p* < .05) and fast-foods at age 5 (*β* = −0.21, *p* < .01) and engaging in fewer sedentary behaviors at age 5 (*β* = −0.14, *p* < .01). Higher nSES at age 5 was associated with consuming less soda/snack foods (*β* = −0.15, *p* < .01) and fast-foods at age 9 (*β* = −0.14, *p* < .01) and engaging in fewer sedentary behaviors at age 9 (*β* = −0.06, *p* < .01). Higher nSES at age 9 was associated with consuming less soda/snack foods (*β* = −0.13, *p* < .001) and fast-foods at age 15 (*β* = −0.16, *p* < .001) and engaging in fewer sedentary behaviors at age 15 (*β* = −0.06, *p* < .01). nSES was not significantly associated with BMIz at ages 5, 9, and 15 (*β*s = −0.08, −0.05, and −0.05, *p* > .05).

Table 2 presents the direct, indirect, and total effects of the relationship between nSES and obesity risk. Soda/snack food intake, fast-food intake, and sedentary behaviors were not significantly associated with BMIz at *α* = 0.05; thus, soda/snack food intake, fast-food intake, and sedentary behaviors did not mediate the association between nSES and BMIz. Soda/snack food intake at age 5 mediated the association between nSES at age 3 and soda/snack food intake at age 9 (*b* = −0.04 [= −0.10 × 0.42]), which explained 23% of the total effect. Fast-food intake at age 5 mediated the association between nSES at age 3 and fast-food intake at age 9 (*b* = −0.07 [= −0.21 × 0.31]; explaining 34% of the total effect), and fast-food intake at age 9 mediated the association between nSES at age 5 and fast-food intake at age 15 (*b* = −0.04 [= −0.27 × 0.17]; explaining 18% of the total effect). Sedentary behaviors at age 9 mediated the association between nSES at age 5 and sedentary behaviors at age 15 (*b* = −0.02 [= −0.13 × 0.15]; explaining 24% of the total effect).

**Table 2:** Direct, Indirect, and Total Effects for the Relationship Between Neighbourhood Socioeconomic Status (nSES) and Unhealthy Diets, Sedentary Behaviours, and Child Body Mass Index *z*-Score (BMIz)

Exposures (E)	Mediators (M)	Outcomes (O)	Pathways	Direct effect		Indirect effect		Total effect	
				*b*	95% CI	*b*	95% CI	*b*	95% CI
nSES at age 3	Soda/snack food intake at age 5	BMIz at age 9	E → M	−0.10^*^	−0.18, −0.01	–	–	−0.10^*^	−0.18, −0.01
			M → O	−0.02	−0.13, 0.09	–	–	−0.02	−0.13, 0.09
			E → O	–	–	−0.09	−0.20, 0.02	−0.09	−0.20, 0.02
nSES at age 3	Fast-food intake at age 5	BMIz at age 9	E → M	−0.21^**^	−0.36, −0.07	–	–	−0.21^**^	−0.36, −0.07
			M → O	0.02	−0.10, 0.07	–	–	0.02	−0.10, 0.07
			E → O	–	–	−0.09	−0.20, 0.02	−0.09	−0.20, 0.02
nSES at age 3	Sedentary behaviors at age 5	BMIz at age 9	E → M	−0.13^**^	−0.21, −0.04	–	–	−0.13^**^	−0.21, −0.04
			M → O	−0.01	−0.07, 0.05	–	–	−0.01	−0.07, 0.05
			E → O	–	–	−0.09	−0.20, 0.02	−0.09	−0.20, 0.02
nSES at age 5	Soda/snack food intake at age 9	BMIz at age 15	E → M	−0.28^**^	−0.43, −0.12	–	–	−0.28^**^	−0.43, −0.12
			M → O	−0.08	−0.21, 0.04	–	–	−0.08	−0.21, 0.04
			E → O	–	–	−0.10	−0.20, 0.01	−0.10	−0.20, 0.01
nSES at age 5	Fast-food intake at age 9	BMIz at age 15	E → M	−0.27^**^	−0.44, −0.09	–	–	−0.27^**^	−0.44, −0.09
			M → O	0.06	−0.06, 0.19	–	–	0.06	−0.06, 0.19
			E → O	–	–	−0.10	−0.20, 0.01	−0.10	−0.20, 0.01
nSES at age 5	Sedentary behaviors at age 9	BMIz at age 15	E → M	−0.13^**^	−0.21, −0.04	–	–	−0.13^**^	−0.21, −0.04
			M → O	−0.01	−0.07, 0.05	–	–	−0.01	−0.07, 0.05
			E → O	–	–	−0.10	−0.20, 0.01	−0.10	−0.20, 0.01
nSES at age 3	Soda/snack food intake at age 5	Soda/snack food intake at age 9	E → M	−0.10^*^	−0.18, −0.01	–	–	−0.10^*^	−0.18, −0.01
			M → O	0.42^***^	0.33, 0.51	–	–	0.42^***^	0.33, 0.51
			E → O	–	–	−0.18^***^	−0.25, −0.11	−0.18^***^	−0.25, −0.11
nSES at age 5	Soda/snack food intake at age 9	Soda intake at age 15	E → M	−0.28^**^	−0.43, −0.12	–	–	−0.28^**^	−0.43, −0.12
			M → O	0.04	−0.05, 0.14	–	–	0.04	−0.05, 0.14
			E → O	–	–	−0.17^***^	−0.23, −0.11	−0.17^***^	−0.23, −0.11
nSES at age 3	Fast-food intake at age 5	Fast-food intake at age 9	E → M	−0.21^**^	−0.36, −0.07	–	–	−0.21^**^	−0.36, −0.07
			M → O	0.31^***^	0.21, 0.41	–	–	0.31^***^	0.21, 0.41
			E → O	–	–	−0.19^***^	−0.29, −0.09	−0.19^***^	−0.29, −0.09
nSES at age 5	Fast-food intake at age 9	Fast-food intake at age 15	E → M	−0.27^**^	−0.44, −0.09	–	–	−0.27^**^	−0.44, −0.09
			M → O	0.17^***^	0.08, 0.26	–	–	0.17^***^	0.08, 0.26
			E → O	–	–	−0.23^***^	−0.32, −0.14	−0.23^***^	−0.32, −0.14
nSES at age 3	Sedentary behaviors at age 5	Sedentary behaviors at age 9	E → M	−0.13^**^	−0.21, −0.04	–	–	−0.13^**^	−0.21, −0.04
			M → O	0.07	−0.09, 0.23	–	–	0.07	−0.09, 0.23
			E → O	–	–	−0.07^**^	−0.12, −0.02	−0.07^**^	−0.12, −0.02
nSES at age 5	Sedentary behaviors at age 9	Sedentary behaviors at age 15	E → M	−0.13^**^	−0.21, −0.04	–	–	−0.13^**^	−0.21, −0.04
			M → O	0.15^**^	0.06, 0.24	–	–	0.15^**^	0.06, 0.24
			E → O	–	–	−0.08^**^	−0.13, −0.02	−0.08^**^	−0.13, −0.02

All analyses were conducted with adjustment for covariates listed in Table 1.

^*^ *p* < .05, ^**^p < .01, ^***^p < .001.

## Discussion

This longitudinal study examines the long-term association between nSES and child weight status and obesity-related unhealthy behaviors from early childhood through adolescence using a large, diverse population sample. On the whole, 17% of children in our sample were overweight or obese at age 3, and this percentage increased to 24% by age 15. The increasing prevalence of childhood obesity from early childhood to adolescence is consistent with past literature [1, 2]. In addition, similar to previous cross-sectional and short-term longitudinal studies [25, 26, 28, 29, 31–33], our results of multivariate analysis showed that nSES has a direct association with unhealthy diets and sedentary behaviors 2–6 years later, after adjusting for parental education level and household income-to-needs ratio. The findings are possible because high-socioeconomic neighborhoods tend to offer more favorable resources that promote healthy behaviors (e.g., access to a park, walkable streets, a safe outdoor environment, and access to healthy food choices) [22, 23, 25]. Given the greater risk for unhealthy behaviors in low-socioeconomic neighborhoods, our findings suggest that nSES needs to be considered in community health policy and practice.

### A Long-lasting Association Between Neighborhood Socioeconomic Status and Unhealthy Behaviors

Our model testing results showed that nSES has a long-lasting impact on shaping children’s unhealthy lifestyles. nSES impacted children’s unhealthy behavioral routines from a young age, and children maintained unhealthy behavioral routines into their adolescent years. Specifically, soda/snack food intake, fast-food intake, and sedentary behaviors at time *t* served as a mediator in the association between nSES at a previous wave (time *t* − 1) and unhealthy behaviors at a subsequent wave (time *t* + 1), except soda/snack food intake and sedentary behaviors at age 9. The results are consistent with past literature reporting that children’s unhealthy lifestyle in early developmental stages tend to persist through later years [47]. This long-lasting nSES impact on children’s unhealthy lifestyles is possibly related to the fact that nSES in early childhood impacts parent and family environments, such as parental rules, parental support, parental feeding style, and healthy food available at home, which persist through adolescence. In particular, during early childhood, parents directly interact with neighbors for their child (e.g., social support from neighbors, sharing social norms within the neighborhood), utilize neighborhood resources for childcare (e.g., access to health-promoting food outlets and activity facilities), and perceive neighborhood atmosphere (e.g., perceptions of social cohesion, crime level). Their interactions with neighborhood environments can determine the quality of parenting and home environments in early childhood [48], which impacts children’s obesity-related behaviors in a long-term period [49–54]. This study has not examined underlying mechanisms by which nSES impacts childhood unhealthy behaviors; thus, further research is needed to examine a mediating mechanism by including parenting, home environments, peer context, school context, and neighborhood built and social environments based on an ecological perspective. An investigation of mediating mechanisms would allow the deconstruction of the nSES impact on childhood unhealthy behaviors and identify actionable mediators to target in future interventions.

Notably, despite strong conceptual arguments for anticipating the mediating role of unhealthy behaviors in nSES effects on childhood obesity, we did not observe a significant mediation effect of unhealthy diets and sedentary behaviors in the association between nSES and child BMIz. The non-significance may be attributed to the increase in unhealthy and sedentary lifestyles due to the increased use of mobile devices, computers, games, and other screen-based technologies in recent decades; thus, these prevalent unhealthy behaviors may not determine BMIz in the last decade. For example, while two past studies using data collected in the 1990s found a significant mediating role of sedentary behaviors in nSES impact on obesity, one study using data collected in the 2010s showed its insignificant mediation effect [35–37]. However, we note that, despite the non-significant mediation, children’s engagement in unhealthy behaviors is critical to intervene because of the adverse effect these behaviors have on physical, behavioral, and mental health in childhood and extending into adulthood [55–57].

### Limitations

The present study has several limitations. First, as mentioned above, this study included only two domains of obesity-related unhealthy behaviors: unhealthy diets and sedentary behavior, thus, is not able to fully explore various behavioral mechanisms that might link nSES and children’s weight status, such as physical activity, fruit and vegetable intake, stress, and sleep. For example, living in disadvantaged neighborhoods is likely to increase psychological stress [58], which can develop into stress-related sleep reactivity, arousal, and emotional dysregulation [59–63] and subsequently discourage healthy lifestyle behaviors [64]. Future research needs to examine a more comprehensive mechanism for understanding the nSES effect on childhood obesity and related behaviors. Second, we relied on self- or primary caregiver-reported measures to capture unhealthy diets and sedentary behavior, which inherently introduce recall bias. Nevertheless, the questions that captured unhealthy diets and sedentary behavior were comprehensive; for example, sedentary behaviors included TV viewing, playing video games, and instant messaging friends (for ages 9 and 15). Third, the measure for sedentary behavior at age 9 was limited to a capped 4-point Likert scale with 2+ hours a day as the largest measure. This capped scale introduces the possibility that variability in this exposure is not sufficiently captured. Fourth, the FFCWS consists of data dating back to the early 2000s, and children’s behavioral patterns may have changed over time and are different from children today. Fifth, although the FFCWS is a national study, it oversampled non-married women and was limited to those in urban areas; hence, it is not representative of the U.S. general population. Also, we excluded children who participated in in-home anthropometric measurements only once during three follow-up studies. Thus, our findings cannot be generalizable to all children and adolescents in the United States. Finally, we did not include peer-related and school-related measures and other neighborhood measures such as neighborhood racial/ethnic segregation, built environments, and social environments. For example, given that non-Hispanic Black and Hispanic children tend to live in lower SES neighborhoods, unfavorable features in low-SES neighborhoods might be related to racial/ethnic segregation, which can lead to insufficient investment in health-promoting resources and services for those in racially segregated neighborhoods [65, 66].

## Conclusions

The present study contributed to our understanding of nSES, unhealthy behaviors, and weight status among children by longitudinally testing the association between time-varying nSES and children’s weight status and obesity-related unhealthy behaviors from early childhood to adolescence in a national sample. Our results indicate that nSES is an important factor to consider, since it can shape unhealthy lifestyles from early childhood through adolescence over and above parental socioeconomic status. Given that existing interventions have targeted disadvantaged children and adolescents based on parental socioeconomic status, our findings highlight the importance of prioritizing children who experience double disadvantage, parental and neighborhood socioeconomic disadvantage, in childhood health policies and practices. Future research is needed to further investigate how neighborhood environments impact childhood obesity and obesity-related behaviors by including comprehensive measures of health behaviors (e.g., healthy eating, moderate-to-vigorous physical activity, sleep, and stress) and neighborhood factors (e.g., access to a park, food retails, walkability, social cohesion, and safety), to inform community health promotion policies and programs.
